# GLP‐1 Receptor Agonists for Treating Alcohol Use Disorder: A Critical Review

**DOI:** 10.1111/acer.70312

**Published:** 2026-05-18

**Authors:** Sean Woo, Grace Zhu, Chrismely Castro, Allen Phuong, Aadita Roy, Christal N. Davis, Henry R. Kranzler

**Affiliations:** ^1^ University of Pennsylvania Philadelphia Pennsylvania USA; ^2^ Perelman School of Medicine University of Pennsylvania Philadelphia Pennsylvania USA; ^3^ Mental Illness Research, Education and Clinical Center Crescenz VAMC Philadelphia Pennsylvania USA

## Abstract

This narrative review evaluates glucagon‐like peptide‐1 receptor agonists (GLP‐1RAs) as potential treatments for alcohol use disorder (AUD), a major public health problem with limited treatment options. We first briefly describe the development of GLP‐1RAs for treating type 2 diabetes and obesity and then focus on recent studies of the drugs' effects on alcohol‐related behaviors, both in animal models and clinical studies. We identified relevant literature by searching for studies of incretin‐based therapies using PubMed, Google Scholar, and clinicaltrials.gov and reviewing the reference lists of published reviews. We limited our discussion of preclinical studies of alcohol‐related effects to those that provide insights into the mechanism of effects. Clinical studies comprised both observational studies, including those using electronic health records and other real‐world data, and randomized controlled clinical trials, including studies completed or in progress. Of the clinical studies, we include original, peer‐reviewed research published in English and excluded case reports, commentaries, and preprints. This yielded a total of 13 preclinical studies, 14 clinical studies, 4 published interventional trials, and 19 interventional trials in progress or completed but unpublished. We found convergent evidence from animal and human studies that GLP‐1RAs reduce alcohol consumption and improve alcohol‐associated outcomes. Several randomized controlled trials (RCTs) in progress aim to test the effects of both established and novel compounds, and different drug formulations and combinations on alcohol consumption. Additional RCTs are needed to establish the efficacy of GLP‐1RAs for treating AUD, with mechanistic studies needed to elucidate more fully their mode of action in reducing alcohol consumption.

## Graphical abstract

This narrative review of the development of GLP‐1 receptor agonists provides a foundation for both preclinical and clinical research on their use for treating alcohol use disorder (AUD). Both observational studies using real‐world data and randomized controlled clinical trials support the potential utility of these medications for treating AUD.

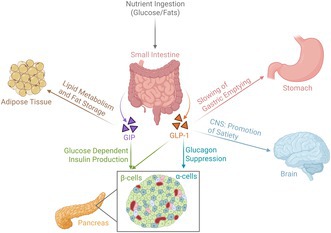

## 1 Introduction

**FIGURE 1:**
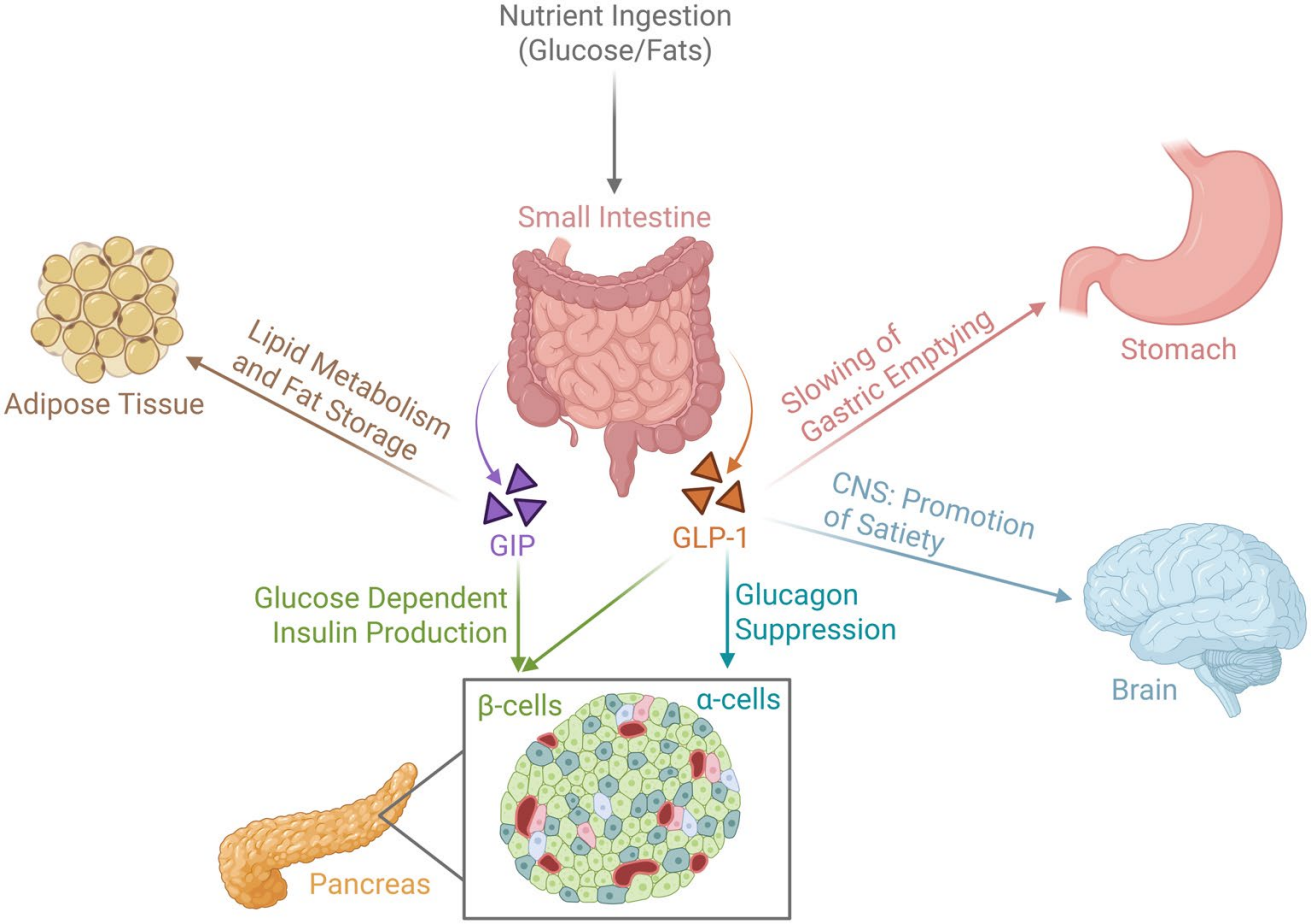
Illustration of the role of gastrointestinal hormones on nutrient ingestion and metabolism.

Incretins—gastrointestinal (GI) hormones of the glucagon superfamily—play a crucial role in glucose homeostasis. The two primary incretin hormones, glucagon‐like peptide‐1 (GLP‐1) and glucose‐dependent insulinotropic polypeptide (GIP), are secreted in the small intestine in response to the ingestion of nutrients, particularly glucose and fats. Both hormones stimulate pancreatic β‐cells to produce insulin in a glucose‐dependent manner, providing precise glycemic control. In addition to boosting insulin secretion, GLP‐1 slows gastric emptying and suppresses glucagon release, which, in combination with its effects on the central nervous system, promotes satiety. GIP primarily enhances insulin secretion but also influences lipid metabolism and fat storage. Incretin‐mimetics, medications that replicate the effects of GLP‐1 and GIP, are now widely used medications for diabetes management and weight loss (Zheng et al. 2024); Figure 1 illustrates the actions of GLP‐1 and GIP. Glucagon, a non‐incretin GI peptide secreted by the pancreas, increases blood glucose concentration by stimulating glycogenolysis and gluconeogenesis, counteracting the effects of insulin. Some GLP‐1RAs are also agonists at the glucagon receptor, which may augment their therapeutic effects.

### 1.1 Development of Incretins for Treating T2D and Obesity/Overweight

Early studies showed that GLP‐1 strongly stimulates insulin secretion while inhibiting glucagon release, making it a promising candidate for the treatment of type 2 diabetes (T2D). This discovery restored interest in incretin‐based therapies, leading to the development of GLP‐1 receptor agonists (GLP‐1RAs) as therapeutic options for T2D and, subsequently, obesity (Zheng et al. 2024). This was accompanied by the development of GLP‐1RAs with agonist or antagonist effects at other GI peptide receptors (e.g., the GIP receptor) that contribute to the drugs' efficacy in treating both T2D and obesity.

In 2005, immediate‐release exenatide, administered twice daily subcutaneously, was the first GLP‐1RA approved by the Food and Drug Administration (FDA) for treating T2D. In 2010, liraglutide was approved as a daily injection for treating T2D, and in 2014, it became the first GLP‐1RA approved to treat obesity. In 2012, extended‐release exenatide was the first GLP‐1RA approved for weekly use in treating T2D, and in 2014, dulaglutide was also approved for weekly administration.

In a series of multicenter studies, semaglutide showed robust efficacy in treating T2D and causing weight loss greater than with all comparators evaluated (Aroda et al. 2019). In 2017, semaglutide was approved for treating T2D. Following a series of studies on weight loss, it was approved in 2021 for that indication (Moiz et al. 2024). Over the next 2 years, the number of semaglutide prescriptions increased from < 500,000 to > 2.5 million (Scannell et al. 2024). Tirzepatide, an agonist at both the GLP‐1 and GIP receptors, was approved by the FDA in 2022 to treat T2D, in 2023 obesity, and in 2025 obstructive sleep apnea.

Several real‐world studies or clinical trials have directly compared the efficacy of semaglutide and tirzepatide in treating T2D or obesity. A cohort study of the two medications in a propensity score‐matched population of 18,386 individuals using electronic health record (EHR) data measured weight loss at 3, 6, and 12 months of treatment (Rodriguez et al. 2024). Tirzepatide‐treated patients were significantly more likely than those treated with semaglutide to achieve weight loss and had greater weight reductions at all three timepoints, while rates of GI adverse events were similar between the medications. A phase 3b trial of 751 adults with obesity and without T2D compared the efficacy and safety of the two drugs at their maximum tolerated dosage, which for tirzepatide was 10 mg or 15 mg and for semaglutide was 1.7 mg or 2.4 mg. After 72 weeks, tirzepatide treatment reduced body weight by nearly 50% more than semaglutide (Aronne et al. 2025). Findings from these studies support the greater efficacy of tirzepatide than semaglutide both in exerting glycemic control and in weight reduction.

Other multiple‐action incretin‐based drugs currently in development with potential utility in treating AUD include mazdutide, maridebart cafraglutide, survodutide, pemvidutide, and retatrutide (Dolgin 2025). Mazdutide (a GLP‐1 and glucagon dual agonist) produced dose‐dependent mean reductions in body weight from baseline to end of treatment in a 24‐week, phase 2 clinical trial in 248 patients with overweight or obesity and without T2D (Ji et al. 2023). In a phase 2 clinical trial of survodutide—also a GLP‐1RA and glucagon receptor agonist—among 387 patients with pre‐obesity or obesity without T2D, those who received the drug lost significantly more weight than those in the placebo group (Le Roux et al. 2024).

Maridebart cafraglutide (MC), a once‐monthly, dual‐action GLP‐1RA agonist and GIP receptor antagonist, was evaluated in mice and obese monkeys and in a phase 1 clinical trial in 75 individuals with obesity and without T2D (Veniant et al. 2024). In preclinical testing, MC reduced both body weight and blood glucose levels. In the clinical study, the drug dose‐dependently reduced body weight, with the effects persisting for up to 150 days posttreatment. A 52‐week, phase 2 study of MC randomized 592 individuals with obesity (465 without T2D and 127 with T2D) to receive MC at one of three dose levels or placebo every 4–8 weeks (Jastreboff et al. 2025). All three groups treated with MC showed significantly greater weight loss than the placebo group. In a 48‐week phase 2 study, 338 participants with obesity and without T2D were randomly assigned to receive subcutaneous retatrutide—a triple‐receptor agonist drug—at one of four dose levels or placebo weekly for 48 weeks (Jastreboff et al. 2023). The decrease in body weight at 48 weeks was dose proportional in the retatrutide groups (which at the highest dose level was a loss of 24.2% of body weight) and greater than with placebo.

Another drug in development combines cagrilintide—a long‐acting analog of amylin that helps to regulate appetite and slow gastric emptying—with semaglutide. In a small, phase 2 trial of individuals with T2D, the combination drug reduced HbA1c levels more than either drug alone (Frias et al. 2023). In a phase 3a, 68‐week, multicenter RCT, 3417 participants with overweight or obesity without T2D received the combination of semaglutide 2.4 mg and cagrilintide 2.4 mg, semaglutide 2.4 mg alone, cagrilintide 2.4 mg alone, or placebo (Garvey et al. 2025). The mean reduction in body weight from baseline to week 68 in the cagrilintide–semaglutide group was significantly greater than with either active drug alone or placebo.

In summary, the role of GLP‐1RAs in managing T2D and obesity is firmly established, with multiple drugs in the class FDA approved for one or both of these indications. The robust findings have sparked interest in using GLP‐1RAs to treat other conditions, resulting in the approval of tirzepatide as therapy for obstructive sleep apnea (Malhotra et al. 2024) and semaglutide for treating chronic renal disease (Perkovic et al. 2024). Because GLP‐1RAs modify reward‐related pathways in the brain, they may also be efficacious in treating substance use disorders (SUDs), where reward‐related effects appear to mediate the development and maintenance of the disorders (Hernandez and Schmidt 2019; Egecioglu et al. 2013b, 2013a). In the review that follows, we focus on the growing literature on the effects of GLP‐1RAs in reducing alcohol use, first in animal models, then in retrospective analyses of real‐world data from EHRs, and finally in prospective RCTs in individuals with harmful drinking or AUD. A key contribution of this article is that it covers exhaustively the rapidly evolving field of clinical studies of GLP‐1RAs for treating harmful alcohol use and AUD, including those for which results are not yet published.

## 2 Methods

To identify articles relevant to the review, we conducted literature searches on February 1, 2025, July 1, 2025, and March 21, 2026. We searched listings on PubMed, Google Scholar, and clinicaltrials.gov of studies of incretin‐based therapies, including GLP‐1RAs, published up to March 21, 2026. We also used a recent review of articles on the use of GLP‐1RAs for treating substance use disorders listed on clinicaltrials.gov to ensure complete coverage of those studies (Patil et al. 2026). The aim of the searches was to (1) identify the published literature on single‐, dual‐, and triple‐action GLP‐1 drugs that have been tested for treating overweight and obesity, and (2) exhaustively review the drugs' use in treating AUD. Whereas immediately prior to publication Klausen et al. (2026) published results from a completed study, we now describe that study fully in the text.

For background information on the use of incretin‐based therapies, search terms included “GLP‐1 AND obesity,” “semaglutide AND cardiovascular outcomes,” “tirzepatide AND weight loss,” “GLP‐1RAs AND opioids,” “dual‐agonist,” “triple‐agonist,” and “triple incretin AND clinical trial.” The literature search for effects on alcohol consumption in animal models and clinical studies was conducted using (“AUD” OR “Alcohol Use Disorder” OR “Alcoholism”) AND (“GLP‐1” OR “Glucagon‐Like Peptide‐1 Receptor Agonists” OR semaglutide OR liraglutide OR dulaglutide OR exenatide OR lixisenatide OR tirzepatide OR brenipatide OR retatrutide OR mazdutide) and included filters to exclude preprints.

We chose these terms to capture experimental studies, observational studies (including those using electronic health records and other real‐world data), and interventional trials. We limited reports to original, peer‐reviewed research published in English, excluding case reports, commentaries, and preprints. We also excluded studies of exendin‐4 because it is not available for use in humans (see Zheng et al. 2025 for a discussion of exendin‐4 studies). This identified 167 studies, including 13 preclinical studies selected to illustrate mechanisms of potential relevance to treating AUD. We identified 14 observational studies and 4 interventional trials, representing all studies of GLP‐1RAs for treating AUD. We also searched trials listed on clinicaltrials.gov as of March 21, 2026, using the terms (“AUD” OR “Alcohol Use Disorder” OR “Alcoholism”) AND (“GLP‐1” OR “Glucagon‐Like Peptide‐1 Receptor Agonists” OR semaglutide OR liraglutide OR dulaglutide OR exenatide OR lixisenatide OR tirzepatide OR brenipatide OR retatrutide OR mazdutide). This yielded 31 trials, 11 of which were excluded because they investigated an unrelated comorbid condition or did not directly investigate the use of a GLP‐1RA for treating AUD, leaving a total of 19 clinical trials in progress or completed and unpublished and one recently published study included here.

## 3 Results

### 3.1 PreClinical Studies of the Effects of GLP‐1RAs on Alcohol Use

Here, we provide a selective review of preclinical studies of the effect of GLP‐1RAs on alcohol consumption and potential mediators of this effect, such as changes in reward processing, stress, and cognitive function (Bruns Vi et al. 2024). For more extensive reviews of the preclinical literature on the alcohol‐related effects of GLP‐1RAs, we refer the reader to two recently published, comprehensive reviews (Zheng et al. 2025; Yammine et al. 2025).

Semaglutide dose‐dependently decreased binge‐like alcohol drinking in both mice and rats and alcohol consumption in alcohol‐dependent rats, supporting its utility in reducing drinking across species and at different levels of drinking intensity (Chuong et al. 2023). Consistent with these findings, Aranas, Edvardsson, et al. (2023) found that acute and repeated administration of semaglutide to both male and female rats reduced alcohol intake and prevented relapse‐like drinking. The effects of semaglutide in reducing alcohol intake in rats was not augmented by combining it with the anti‐smoking agents varenicline or bupropion (Aranas, BLID Skoldheden, and Jerlhag 2023). Liraglutide (50 nmol/kg) also reduced alcohol consumption and preference in a 2‐bottle choice paradigm and a deprivation‐induced relapse model. Among mice subjected to a period of forced withdrawal, those treated with liraglutide performed better on measures of learning and memory and showed less anxiety than untreated mice (Liu et al. 2024). Most recently, tirzepatide dose‐dependently reduced alcohol consumption, preference, and relapse‐like drinking in both male and female rats (Edvardsson et al. 2026). Dulaglutide (0.05 or 0.1 mg/kg) treatment administered either for 5 or 9 weeks, reduced alcohol intake and preference in both male and female rats (Vallof et al. 2020). Treatment outcomes with dulaglutide also depended on how it was combined with the amylin receptor agonist sCT, a drug that also reduces alcohol consumption. While dulaglutide (0.1 mg/kg) alone reduced alcohol intake, simultaneous treatment with sCT initially reduced alcohol intake but then led to tolerance. However, administration of sCT after dulaglutide reduced alcohol consumption in both male and female rats (Aranas et al. 2024). Extending these findings (Windram et al. 2025), found that semaglutide, tirzepatide, and retatrutide reduced the interoceptive effects of alcohol, a potential mechanism contributing to incretin‐based modulation of alcohol‐related behaviors.

Although the behavioral effects of GLP‐1RAs have been studied extensively in rodents, only two such studies have been conducted in nonhuman primates, with both conducted in vervet monkeys selected for voluntary alcohol drinking. In the first study, animals treated with liraglutide consumed significantly less alcohol with no change in water intake, with a similar but weaker effect also seen with exenatide treatment (Thomsen et al. 2019). In the second study, alcohol consumption was measured for 10 days at baseline, followed by administration of escalating doses of semaglutide (up to 0.05 mg/kg) or vehicle subcutaneously twice weekly for 2 weeks. Semaglutide‐treated monkeys consumed significantly less alcohol compared both to baseline drinking and to vehicle‐treated monkeys with no difference in water intake (Fink‐Jensen et al. 2025).

The convergent behavioral findings across GLP‐1RAs and species point to shared mechanistic pathways, centered on mesolimbic dopamine suppression. Fluorescently labeled semaglutide was detected in the nucleus accumbens (NAc) of alcohol‐drinking rats, which in male mice reduced the dopamine increase associated with alcohol consumption, enhanced the dopamine metabolites DOPAC and HVA in the NAc, and increased the expression of the dopamine catabolizing enzymes COMT and MAOA (Aranas, Edvardsson, et al. 2023). Thus, semaglutide‐induced reductions in alcohol drinking appear to be mediated by reductions in alcohol‐induced reward and related NAc‐dependent mechanisms. Liraglutide (0.1 mg/kg) also significantly attenuated the alcohol‐induced increases in NAc dopamine levels compared to vehicle controls (Vallof et al. 2016). Tirzepatide similarly reduced alcohol‐induced NAc dopamine release and the associated increase in dopamine metabolites (Edvardsson et al. 2026). Dulaglutide also reduced ethanol intake and preference in both male and female rats, though its impact on this measure was greater among males and, after discontinuation of the drug, the decrease persisted in males, but not females. Nine weeks of dulaglutide treatment also differentially influenced monoaminergic signaling in reward‐related areas of male and female rats, reflecting potential sex differences in the mechanism of GLP‐1RA effects on alcohol consumption (Vallof et al. 2020).

GABAergic transmission and synaptic integrity have also been implicated in the mechanism of the alcohol‐related effects of GLP‐1RAs. Semaglutide enhanced GABA release in alcohol‐naive rats through increased spontaneous inhibitory postsynaptic current frequency in neurons of the central amygdala and infralimbic cortex. However, in alcohol‐dependent rats, overall GABA transmission was not affected (Chuong et al. 2023). Additionally, dendritic spine density in the medial prefrontal cortex and hippocampus, and related measures of synaptic function reduced by alcohol withdrawal, were restored to normal levels by liraglutide (Liu et al. 2024). Finally, tirzepatide produced synaptic depression in the lateral septum (Edvardsson et al. 2026).

A critical mechanistic question is whether these effects require direct, pharmacological GLP‐1R engagement or can they be achieved by raising endogenous GLP‐1 levels. Marty et al. (2020) examined the direct agonist effect on GLP‐1R by comparing semaglutide and liraglutide treatment in Wistar rats given access to alcohol. Semaglutide reduced alcohol consumption on the day of injection, while liraglutide increased water intake with a concomitant, longer lasting reduction in alcohol intake. Notably, administration of sitagliptin, a DPP‐4 inhibitor (DPP‐4I) that reduces blood glucose levels by increasing the concentration of GLP‐1, did not affect alcohol consumption. Thus, augmenting endogenous GLP‐1 signaling does not meaningfully contribute to the behavioral outcomes produced by pharmacological activation of the receptor. Moreover, Farokhnia et al. (2025) found that, in both mouse and rat models, where alcohol consumption was reduced by semaglutide treatment, the DPP‐4Is linagliptin and omarigliptin had no effect on drinking behavior. Together, these findings suggest that DPP‐4Is produce physiologically constrained increases in GLP‐1 that are insufficient to engage the neural reward systems underlying alcohol consumption, for which direct pharmacological activation of GLP‐1Rs is required.

In summary, preclinical studies of GLP‐1RAs consistently show that they reduce alcohol consumption, providing a clear rationale for clinical studies of these drugs' therapeutic effects in individuals with AUD. The studies also implicate specific neuropharmacological mechanisms, principally implicating dopaminergic reward pathways that underlie the behavioral effects.

### 3.2 Clinical Studies of GLP‐1RAs on Alcohol Use

#### 3.2.1 Studies Using EHR Data or Implemented via Social Media

Numerous retrospective analyses of data from EHRs or social media support the utility of GLP‐1RAs for treating AUD (see Table 1). A large retrospective cohort study using EHR data examined semaglutide's effects on the incidence of AUD in multiple real‐world patient populations, including those with obesity only (*n* = 83,825), obesity and a history of AUD (*n* = 4254), and T2D only (*n* = 598,803). Semaglutide treatment was associated with a significantly lower risk of developing AUD in all three patient populations, though the study's 12‐month follow‐up could not evaluate its long‐term effects on AUD risk. Whereas the dosage of the drug also differed between groups (2.4 mg for obesity and 0.5–1 mg for T2D), the outcomes were confounded with diagnosis (Wang et al. 2024). Palzes et al. 2025, in an analysis of EHR data from 1214 GLP‐1RA‐treated and 1063 untreated, propensity‐score matched individuals without AUD, found a greater reduction in alcohol intake among the GLP‐1‐treated group than the unexposed group (1.81 drinks/week vs. 1.38 drinks/week, respectively), a nonsignificant difference‐in‐difference (DiD) of −0.43, 95% CI −0.87 to 0.01. In one of multiple subanalyses, low‐risk drinkers (~93% of the cohort) treated with a GLP‐1RA showed a modest but significant reduction in alcohol consumption (DiD = −0.32, 95% CI −0.64 to −0.01). However, in a very large EHR cohort (> 100 million patients, *n* = 817,309 with AUD), GLP‐1RA prescriptions were associated with significantly lower rates of alcohol intoxication in AUD patients (adjusted incidence rate ratio (aIRR) = 0.50 [0.40–0.63]). A Danish national cohort study compared individuals newly prescribed either a GLP‐1RA (*n* = 38,454) or a DPP‐4I (*n* = 49,222) across an 8‐year follow‐up period. The study measured alcohol‐related events, defined as hospital contact with AUD as the main diagnosis, registered treatments for AUD, or the purchase of any medication used to treat AUD, such as chlordiazepoxide, typically prescribed for treating alcohol withdrawal. Users of GLP‐1RAs had substantially lower rates of alcohol‐related events (hazard ratio [HR] = 0.46), supporting further investigation of the use of GLP‐1RAs for preventing such alcohol‐related events (Wium‐Andersen et al. 2022).

**TABLE 1:** Summary of EHR‐ or Social‐Media‐Based Studies of GLP‐1RAs and AUD‐related outcomes.

Project title	Authors year	Condition/Population	Study period location	Intervention follow‐up time (s)	Primary outcome
		BMI target			
		Sample size			
Use of GLP‐1 receptor agonists and subsequent risk of alcohol‐related events. A nationwide register‐based cohort and self‐controlled case series study	Wium‐Andersen et al. 2022	Users of GLP‐1RAs and DPP‐4 inhibitors *N* = 38,454 (GLP‐1RAs) *N* = 49,222 (DPP‐4 inhibitors)	2009–2017 Denmark	Semaglutide vs. DPP‐4 inhibitors Median of 4.1 years	Alcohol‐related events
Semaglutide and tirzepatide reduce alcohol consumption in individuals with obesity	Quddos et al. 2023	Current alcohol drinkers identified via social media BMI: ≥ 30 kg/m^2^ *N* = 153	Dates not specified United States	Semaglutide 0.25–2.4 mg vs. Tirzepatide 0.5–15 mg vs. control ≥ 30 days	AUDIT score and alcohol consumption collected remotely
Associations of semaglutide with incidence and recurrence of alcohol use disorder in real‐world population	Wang et al. 2024	Obesity BMI: ≥ 30 kg/m^2^ *N* = 83,825 T2D BMI: ≥ 30 kg/m^2^ *N* = 598,803	June 2021–December 2022 United States	Semaglutide 2.4 mg weekly vs. Non‐GLP‐1RA anti‐obesity medications Semaglutide 0.5–1.0 mg weekly vs. Non‐GLP‐1RA anti‐obesity medications 12 months	Risk for incident and AUD relapse
Glucagon‐like peptide‐1 receptor agonists, but not dipeptidyl peptidase‐4 inhibitors, reduce alcohol intake	Farokhnia et al. 2025	GLP‐1RA recipients, DPP‐4I recipients, or unexposed individuals *N* = 14,130 vs. 12,398 (GLP‐1RA vs. unexposed) *N* = 44,498 vs. 40,938 (DPP‐4I vs. unexposed) *N* = 11,863 vs. 11,145 (GLP‐1RA vs. DPP‐4I)	2008–2023 U.S. Department of Veterans Affairs	GLP‐1RA vs. DPP‐4I vs. control Maximum of 2 years	AUDIT‐C score
Association of glucagon‐like peptide‐1 receptor agonist with progression to liver cirrhosis and alcohol‐related admissions in patients with alcohol use disorder and diabetes: A retrospective cohort study	Al‐Moussally et al. 2025	AUD and T2D *N* = 9965 (GLP‐1RA) *N* = 19,688 (DPP4i)	2006–2021 U.S. Veterans health administration	GLP‐1RA vs. DPP‐4I From first prescription fill to study end (September 30, 2021), death, or initiation of a GLP‐1RA in the DPP4i group	Progression to cirrhosis and alcohol‐related hospitalization
Repurposing semaglutide and liraglutide for alcohol use disorder	Lahteenvuo et al. 2025	AUD *N* = 227,866	January 2006–December 2023 Sweden	GLP‐1 agonists vs. control Median of 8.8 years	AUD hospitalization
Mapping the effectiveness and risks of GLP‐1 receptor agonists	Xie et al. 2025	T2D *N* = 215,970 (GLP‐1RAs) *N* = 159,465 (sulfonylureas) *N* = 117,989 (DPP‐4 inhibitors) *N* = 258,614 (SGLT2 inhibitors) *N* = 536,068 (pooled comparator group) *N* = 1,203,097 (usual care group)	October 2017–December 2023 U.S. Department of veterans affairs	GLP‐1RA vs. sulfonylurea vs. DPP‐4I vs. SGLT2 inhibitors vs. control vs. usual care Median of 3.68 years	Incident GLP‐1RA use and the risk of 175 associated beneficial or adverse outcomes
The association between glucose‐dependent insulinotropic polypeptide and/or glucagon‐like peptide‐1 receptor agonist prescriptions and substance‐related outcomes in patients with opioid and alcohol use disorders: A real‐world data analysis	Qeadan et al. 2025	AUD *N* = 5621 (GLP‐1RAs) *N* = 811,688 (untreated)	January 2014–August 2022 United States	GIP/GLP‐1RAs vs. untreated Up to 24 months	Alcohol intoxication
Glucagon‐like peptide‐1 receptor agonists and alcohol use: a real‐word observational study in a large, integrated health care system	Palzes et al. 2025	Users of GLP‐1RAs *N* = 1214 (GLP‐1RAs) *N* = 1063 (untreated)	June 2013–June 30, 2023 United States	GLP‐1RAs vs. untreated 12 months	Difference‐in‐differences of average number of drinks per week
GLP‐1 receptor agonists and substance use disorders in older adults with type 2 diabetes: A target trial emulation	Dai et al. 2025	GLP‐1RA recipients, DPP‐4I recipients, or SGLT2 inhibitor recipients *N* = 2310 vs. 2310 (GLP‐1RAs vs. SLGT2 inhibitors) *N* = 2460 vs. 2460 (GLP‐1RAs vs. DPP4I)	January 2016–December 2020 United States	GLP‐1RAs vs. DPP‐4I vs. SGLT2 inhibitors Up to 4 years	SUD and AUD hospitalization
Genetically modeled GLP1R and GIPR agonism reduce binge drinking and alcohol‐associated phenotypes: a multi‐ancestry drug‐target Mendelian randomization study	Reitz et al. 2025	*N* = 666,978 (European) *N* = 90,852 (East Asian) *N* = 8078 (African)	United Kingdom (UK Biobank; UK Biobank Pan‐UKB release); United States (Million Veterans Program); Japan (Biobank Japan)	Genetic proxies for GLP‐1/GIP signaling	Problematic alcohol use
Blood phosphatidylethanol measurements indicate GLP‐1 receptor stimulation causes delayed decreases in alcohol consumption	Jensen et al. 2025	AUD BMI ≥ 30 kg/m^2^ *N* = 30	August 2017–October 2019 Denmark	GLP‐1RA vs. placebo 4, 12, 20, and 26 weeks	PEth levels
Real‐world alcohol use disorder outcomes in patients with concurrent metabolic dysfunction: glp‐1 receptor agonists versus FDA‐approved AUD medications	Gougol et al. 2026	MedD and AUD BMI ≥ 25 kg/m^2^ *N* = 274 (GLP‐1RAs) *N* = 1272 (Naltrexone) *N* = 232 (Acamprosate) *N* = 168 (Disulfiram)	January 2017–January 2025 United States	GLP‐1RAs vs. Naltrexone vs. Acamprosate vs. Disulfiram At least 12 months	Incidence of AUD relapse
Relative efficacy of GLP‐1 and GLP‐1/GIP receptor agonists in the prevention of alcohol‐use disorders using a target trial emulation approach	Henney et al. 2026	T2D *N* = 7165 vs. 7165 (Tirzepatide vs. DPP4I) *N* = 20,198 vs. 20,198 (Semaglutide vs. DPP4I) *N* = 6565 vs. 6565 (Liraglutide vs. DPP4I) *N* = 19,061 vs. 19,061 (Dulaglutide vs. DPP4I)	May 2022–November 2023 United States	GLP‐1RAs vs. DPP4I 18 months	AUD diagnosis

Abbreviations: AUD, alcohol use disorder; AUDIT, Alcohol Use Disorders Identification Test; AUDIT‐C, AUDIT‐Consumption Score; BMI, body mass index; DPP‐4I, dipeptidyl peptidase‐4 inhibitor; GLP‐1RA, glucagon‐like peptide‐1 receptor agonists; MetD, Metabolic dysfunction; PEth, phosphatidylethanol; SGLT2, sodium‐glucose cotransporter 2; SUD, substance use disorder; T2D, type‐2 diabetes.

Quddos et al. 2023 enrolled 153 current drinkers in a study conducted remotely using social media. Participants completed assessments of their alcohol consumption before and during their current medication regimen. Participants who were receiving semaglutide (*n* = 56) or tirzepatide (*n* = 50) consumed significantly fewer drinks and had lower odds of binge drinking and lower Alcohol Use Disorders Identification Test (AUDIT) scores than those on neither medication (*n* = 47). However, the study sample was small, self‐selected, restricted to obese patients most of whom were female and white. In addition, there was no random assignment to treatment conditions and drinking estimates were limited to self‐reports, which are susceptible to misrepresentation or inaccurate recall.

Numerous retrospective studies have compared the effects on alcohol‐related outcomes of GLP‐1RAs with FDA‐approved treatments for AUD. Farokhnia et al. 2025 used the three‐item AUDIT‐Consumption measure (AUDIT‐C), obtained for the period 2008–2023 from the Department of Veterans Affairs' EHR, to compare alcohol consumption among propensity score‐matched individuals treated with a GLP‐1RA, a DPP‐4I, or neither drug class. GLP‐1RA recipients reported a greater reduction in AUDIT‐C scores than unexposed individuals and DPP‐4I recipients. There were no differences between DPP‐4I recipients and unexposed individuals. The presence of an AUD diagnosis was associated with a greater drinking reduction in GLP‐1RA‐treated patients than either unexposed or DPP‐4I‐treated patients. Baseline hazardous drinking was also associated with a greater reduction among GLP‐1RA‐treated individuals than unexposed ones. However, another retrospective propensity score‐matched cohort study of veterans with AUD who received treatment with a GLP‐1RA (*n* = 9965) or a DPP‐4I (*n* = 19,688) showed no effect of either drug on liver cirrhosis progression or hospital admissions due to alcohol, relatively uncommon outcomes with wide confidence intervals (CIs) that limit the interpretation of the findings (Al‐Moussally et al. 2025). Lastly, in a comparison of GLP‐1RAs with three widely used treatments for T2D—sulfonylureas, DPP‐4Is, and sodium‐glucose cotransporter‐2 inhibitors (SGLT2Is)—in more than 2 million patients with T2D in the VA healthcare system, GLP‐1RAs were linked to altered risk for 175 diseases and conditions. These included reduced risk of SUDs, including AUD (HR = 0.89), which remained significant after adjustment for demographic and clinical confounders (Xie et al. 2025).

Gougol et al. 2026 compared the effects of GLP‐1RAs with FDA‐approved AUD medications in a retrospective cohort study of patients with metabolic dysfunction and AUD. Treatment with a GLP‐1RA (*n* = 274) was associated with a lower rate of AUD relapse (incidence rate ratio (IRR) = 0.55, 95% CI = 0.42–0.73; *p* < 0.01) than exposure to naltrexone (*n* = 1272), acamprosate (*n* = 232), or disulfiram (*n* = 168). An 8‐year retrospective cohort study compared the effects of the GLP‐1RAs exenatide, liraglutide, dulaglutide, and semaglutide with those of disulfiram, acamprosate, or naltrexone on AUD‐related hospitalizations (Lahteenvuo et al. 2025). Treatment with a GLP‐1RA, particularly semaglutide and liraglutide, was associated with a lower risk of hospitalization than seen with the medications approved for treating AUD. Semaglutide was more effective than liraglutide in reducing hospitalization risk. This suggests that semaglutide is a better candidate than liraglutide for treating AUD due both to its greater efficacy and weekly (rather than daily) administration.

Target trial emulation approaches have yielded mixed results for the efficacy of GLP‐1RAs in the prevention and treatment of AUD. In a target‐trial emulation study using real‐world EHR data (> 120 million patients) of T2D patients without AUD, tirzepatide (*n* = 7165) and semaglutide (*n* = 20,198) were associated with significantly lower incident AUD than DPP4Is (HR = 0.47 [0.29–0.75] and 0.68 [0.52–0.89], respectively). However, liraglutide and dulaglutide were not significantly associated with these effects. When directly compared, tirzepatide reduced AUD risk more than liraglutide (HR = 0.47 [0.24–0.92]), consistent with prior findings that some GLP‐1‐based therapies provide greater protective effects than others (Henney et al. 2026). In a target trial emulation study using Medicare data, GLP‐1RA use among older adults with T2D (*n* = 4920) was associated with a lower rate of hospitalization for an SUD than treatment with a DPP4I (HR = 0.76 [0.67–0.86]). However, for AUD specifically, the reduction was not statistically significant (HR = 0.76 [0.53–1.08]) and no differences were observed when comparing GLP‐1RA treatment with SGLT2I treatment (*n* = 4620) (Dai et al. 2025).

Genetic epidemiological approaches have also been applied to study the relationship between GLP‐1RAs and AUD. Using Mendelian randomization, genetic proxies related to GLP‐1/GIP signaling were associated with reduced problematic alcohol use and binge drinking (β = −0.44, 95% CI = −0.72, −0.15, *p* = 2.42 × 10^−3^ in a primary sample and β = −0.13, 95% CI = −0.22, −0.04, *p* = 0.0058 in a replication sample) and lower odds of heavy drinking (OR = 0.62 [0.45–0.85]). In contrast, effects on other SUDs (tobacco, cannabis, opioid) were largely null. Although this suggests a more specific impact on alcohol‐related behaviors (Reitz et al. 2025), there is a growing literature that supports the potential utility of GLP‐1RAs for treating other SUDs, which we briefly describe below.

Despite a growing body of clinical epidemiological evidence of the beneficial effects of GLP‐1RAs for treating AUD, RCTs are the “gold standard” of clinical pharmacology. Thus, RCTs are needed to establish the therapeutic efficacy and ultimately to obtain FDA approval of GLP‐1RAs for treating AUD. Below, we review completed, ongoing, and planned RCTs of these medications.

#### 3.2.2 Completed Clinical Trials

The first RCT of a GLP‐1RA for treating AUD (see Table 2) was a 26‐week, placebo‐controlled study of weekly exenatide 2 mg in 127 treatment‐seeking patients who also underwent functional magnetic resonance imaging (fMRI) brain scans (Klausen, Jensen, et al. 2022). Although exenatide did not significantly reduce the number of heavy drinking days (HDDs) more than placebo, it attenuated the fMRI signal in the ventral striatum and septum during alcohol cue reactivity. In a secondary analysis of study outcomes, exenatide reduced HDDs and total alcohol intake in the subset of patients (*n* = 30) who were obese (BMI > 30 kg/m^2^). Analysis of this subgroup also showed a significantly greater reduction in phosphatidylethanol concentration (a biomarker of alcohol consumption) at week 26 in the exenatide group than the placebo group, with no significant differences at earlier time points (Jensen et al. 2025). Together, these findings suggest that the greatest therapeutic potential of exenatide for AUD is in individuals with obesity and that its effect on alcohol use is time dependent.

**TABLE 2:** Overview of completed clinical trials evaluating GLP‐1RAs' effects on alcohol‐related disorders or smoking.

Publication title	Authors and year of publication	Condition/Population	Study location	Intervention, follow‐up times	Primary outcome
		BMI target			
		Sample size			
Exenatide once weekly for alcohol use disorder investigated in a randomized, placebo‐controlled clinical trial	Klausen, Jensen, et al. 2022	Alcohol dependence BMI ≥ 18.5 kg/m^2^ *N* = 152	Denmark	Exenatide 2 mg weekly sc vs. placebo 26 weeks	Heavy drinking days
Effects of dulaglutide on alcohol consumption during smoking cessation	Probst et al. 2023	Alcohol consumption among smokers in treatment BMI 18–30 kg/m^2^ *N* = 256	Switzerland	Dulaglutide 1.5 mg sc weekly vs. placebo 12 weeks	Standard drinks
Once‐weekly semaglutide in adults with alcohol use disorder: A randomized clinical trial	Hendershot et al. 2025	Moderate AUD Mean BMI = 32 kg/m^2^ *N* = 48 nontreatment seeking	United States	Semaglutide 0.25–1.0 mg sc vs. placebo 9 weeks	Laboratory alcohol responses and consumption, naturalistic alcohol consumption, and weight loss
Once‐weekly semaglutide versus placebo in patients with alcohol use disorder and comorbid obesity: a randomised, double‐blind, placebo‐controlled trial.	Klausen et al. 2026	Moderate‐to‐severe AUD BMI > 30 kg/m^2^ *N* = 108 treatment‐seeking	Denmark	Semaglutide 0.25–2.4 mg sc vs. placebo 26 weeks	Heavy drinking days

Abbreviations: AUD, alcohol use disorder; BMI, body mass index; sc, subcutaneous.

A secondary analysis of findings from a 12‐week, double‐blind RCT of the GLP‐1RA dulaglutide for smoking cessation in 255 patients examined patients' drinking behavior (Probst et al. 2023). Despite having no effect on smoking, treatment with dulaglutide was associated with a 29% reduction in alcohol consumption, which was significantly greater than that in the placebo group.

A phase 2 clinical trial in 48 nontreatment‐seeking adults with AUD who received subcutaneous semaglutide or placebo included both human laboratory and clinical trial components (Hendershot et al. 2025). Before starting treatment, participants completed an alcohol administration session, followed by weekly medication or placebo treatment for 8 weeks. A posttreatment laboratory session was then conducted, followed by a final discharge visit. In the laboratory sessions, participants underwent a standardized alcohol administration paradigm, where they could delay drinking for monetary rewards or consume alcohol at their own pace. Key outcomes included the volume of alcohol consumed and peak breath alcohol concentration (BrAC). The laboratory study showed a medium‐to‐large effect on alcohol consumption, with a lower peak BrAC in the semaglutide‐treated participants than the placebo group. Following the first laboratory session, both weekly alcohol use and smoking were significantly lower in the active treatment arm. Semaglutide reduced the number of drinks per drinking day, the number of HDDs, and alcohol craving and in smokers the number of cigarettes per day more than placebo. The robust effects on drinking measures in this trial, compared to the modest ones in the exenatide trial, suggest that semaglutide is a more effective GLP‐1RA for treating AUD than exenatide. The inclusion of nontreatment‐seeking individuals in the semaglutide trial also suggests that the observed effects of the medication may not depend on participants' motivation to reduce or stop drinking.

A recently published 26‐week trial randomly assigned 108 treatment‐seeking patients (53 women and 55 men) with moderate‐to‐severe AUD and obesity to receive either semaglutide (maximum dosage 2.4 mg/week) or placebo (Klausen et al. 2026). All patients also received cognitive behavioral therapy. Eighty‐one percent of patients completed the full trial. Semaglutide was associated with a 41.1% reduction in the primary outcome—heavy drinking days—from baseline, compared to a 26.4% reduction in the placebo group (*p* = 0.0015), with significant effects on multiple secondary outcomes, including a reduction in PEth. Although more common in the semaglutide group, the most common adverse events, which were gastrointestinal, were generally mild to moderate. Thus, this study complements that of Hendershot et al. (2025) by showing similar robust therapeutic effects of semaglutide in treatment‐seeking participants with moderate‐to‐severe AUD.

Although other clinical trials of GLP‐1RAs for treating AUD have recently been completed, the results have not yet been published or posted on clinicaltrials.gov. An 8‐week trial (NCT05892432) compared the effects of oral semaglutide treatment with placebo in reducing alcohol craving and consumption in 50 individuals with AUD. The dosage of semaglutide was increased from 3 mg/day during the first half to 7 mg/day during the second half of the 8‐week trial. Before the study medication was initiated, participants completed an alcohol cue reactivity task and an fMRI scan, which were repeated at weeks 6 and 8, respectively. A recently completed 12‐week, placebo‐controlled RCT (NCT05891587) assessed the efficacy and tolerability of semaglutide in 80 individuals with AUD. The dosage of semaglutide was increased to a maximum of 1 mg/week. Participants underwent fMRI, functional near‐infrared spectroscopy, and virtual reality experiments at baseline and at the end of treatment. A 26‐week study of semaglutide (NCT05895643) compared the number of HDDs following treatment with either subcutaneous semaglutide at a maximal dosage of 2.4 mg/week with placebo in 108 patients diagnosed with AUD and obesity. Structural MRI and fMRI scans were performed pre‐ and posttreatment to examine neural changes associated with the treatment. A 28‐week trial (NCT06409130), in which 240 patients with alcohol‐related liver disease were enrolled, compares the effects of zalfermin, a long‐acting fibroblast growth factor 21 analog; cagrilintide, an amylin analog; and semaglutide, individually and in combination, on liver damage and alcohol consumption. In addition, one trial (NCT03645408) was terminated due to COVID‐19 hospital‐wide policies that halted recruitment aimed at testing the effect of exenatide on alcohol self‐administration and craving following a priming dose of alcohol.

#### 3.2.3 Ongoing or Planned Clinical Trials

There are multiple ongoing or planned clinical trials or human laboratory studies of semaglutide for treating AUD (see Table 3). A 12 weeks, placebo‐controlled trial (NCT06015893) continues to recruit participants to study the safety and efficacy of subcutaneous semaglutide 2.4 mg/week or the maximal tolerated dosage in 52 participants with hazardous or harmful drinking. The co‐primary outcome measures are drinks per week and the number of severe adverse events. A virtual reality‐based assessment of food preferences and an fMRI scan component augment the clinical trial findings. A 12‐week, open‐label pilot trial that is currently recruiting patients will combine semaglutide with medical management and a clinical pharmacist‐delivered behavioral intervention in 30 individuals with human immunodeficiency virus (HIV) and AUD (NCT07040592). The primary outcomes are adherence (session attendance and medication fills) and safety (as measured by the occurrence of adverse events).

**TABLE 3:** Overview of ongoing or recently completed clinical trials evaluating GLP‐1RAs' effects on AUD, alcohol‐related liver disease, or smoking.

Project title	Investigators	Condition/Population	Trial registration location (s) sponsor	Intervention, follow‐up times	Primary outcome	Status projected start/end^a^
		BMI target				
		Sample size				
Clinical trial of rybelsus (semaglutide) among adults with alcohol use disorder (AUD)	Schacht et al.	AUD BMI: < 25 kg/m^2^ *N* = 135	NCT05892432 USA (Aurora, CO) University of colorado, denver	Oral semaglutide 3–7 mg daily vs. placebo 24 weeks	Alcohol craving and consumption; fMRI cue reactivity	Completed
Effects of NNC0194‐0499, cagrilintide and semaglutide on liver damage and alcohol use	Shah et al.	Alcohol‐related liver disease BMI: < 25 kg/m^2^ *N* = 270	NCT06409130 Global (86 locations) Novo nordisk A/S	Zalfermin, cagrilintide, semaglutide vs. placebo 39 weeks	Liver damage biomarkers; alcohol consumption	Active, not recruiting 2024–2026
GLP‐1 RA on alcohol and metabolic Parameters in obesity and fatty liver disease	Rodrigues et al.	AUD and fatty liver disease BMI: ≥ 35 kg/m^2^ or ≥ 28 kg/m^2^ with weight‐related comorbidities *N* = 64	NCT06546384 Switzerland Insel Gruppe AG, University hospital bern	Semaglutide 0.25–2.4 mg vs. placebo 8 weeks	Alcohol intake; metabolic/liver biomarkers	Not yet recruiting 2025–2027
Semaglutide therapy for alcohol reduction	Leggio et al.	AUD BMI: < 50 kg/m^2^ and ≥ 23 kg/m^2^ *N* = 52	NCT06015893 USA (Baltimore, MD) National institute on drug abuse	Semaglutide 2.4 mg weekly sc vs. placebo 20 weeks	Heavy drinking days and drinks per week	Recruiting 2023–2030
Semaglutide therapy for alcohol reduction—tulsa	Simmons et al.	AUD BMI: < 50 kg/m^2^ and ≥ 25 kg/m^2^ *N* = 80	NCT05891587 USA (Tulsa, OK) Oklahoma State University Center for Health sciences	Semaglutide 0.25–1.0 mg weekly sc vs. placebo 12 weeks	Change in alcohol drinks consumed per week	Completed
Pemvidutide in AUD with Overweight/Obesity	Tomah et al.	AUD BMI: ≥ 25 kg/m^2^ *N* = 100	NCT06987513 USA (12 locations) Altimmune Inc	Pemvidutide 2.4 mg weekly sc vs. placebo 16 weeks	Change in heavy drinking days	Active, not recruiting 2025–2026
A study to evaluate mazdutide compared with placebo in aud	Lilly pharmaceuticals	AUD *N* = 300	NCT06817356 USA (27 locations) Eli lilly and company	Mazdutide sc vs. placebo 36 weeks	AUD‐associated behaviors (TLFB)	Recruiting 2025–2026
Tirzepatide for alcohol use disorder	Hendershot et al.	AUD BMI: ≥ 27 kg/m^2^ *N* = 42	NCT06994338 USA (Los Angleles, CA) University of southern california	Tirzepatide 2.5–5.0 mg weekly sc vs. placebo 8 weeks	Number of heavy drinking days	Recruiting 2025–2026
Study of tirzepatide for recovery and alcohol use management	Suzuki et al.	AUD BMI: ≥ 23 kg/m^2^ *N* = 20	NCT06727331 USA (Boston, MA) Brigham and women's hospital	Tirzepatide 2.5 mg weekly sc vs. placebo 4 weeks	Cue‐induced cravings for alcohol; incidence and severity of adverse events	Recruiting 2025–2026
Effects of tirzepatide on alcohol intake in patients diagnosed with schizophrenia and alcohol use disorder	Fink‐Jensen et al.	AUD and schizophrenia spectrum disorder BMI: ≥ 23 kg/m^2^ *N* = 108	NCT06939088 Denmark Psychiatric centre rigshospitalet	Tirzepatide maximum dose 15 mg sc vs. placebo 26 weeks	Change in heavy drinking days (TLFB)	Recruiting 2026–2028
Off‐label medications for alcohol use disorder among patients with hiv: Pilot study 3 semaglutide	Marconi et al.	AUD and HIV BMI: ≥ 23 kg/m^2^ *N* = 30	NCT07040592 USA (Decatur, GA) Yale university	Semaglutide 12 weeks	Proportion of participants who complete sessions; adherence to medication; number of sessions completed; adverse event reporting	Recruiting 2026–2027
Cessation or reduction of alcohol consumption in veterans: A randomized, double‐blind, placebo‐controlled phase 3 trial to evaluate the efficacy and safety of a glp‐1 receptor agonist semaglutide in U.S. Veterans with alcohol use disorder	Oslin et al.	AUD and veteran BMI: ≥ 21 kg/m^2^ *N* = 622	NCT07218354 USA (Philadelphia, PA) VA Office of research and development	Semaglutide 2.4 mg/week sc or max tolerated dose vs. placebo 28 weeks	Two‐level reduction in the World Health Organization (WHO) risk drinking level	Not yet recruiting 2026–2028
A Study of brenipatide in participants with moderate‐to‐severe alcohol use disorder	Lilly pharmaceuticals	Moderate‐to‐severe AUD *N* = 1100	NCT07219966 Worldwide (118 sites) Eli lilly and company	Brenipatide sc vs. placebo 56 weeks	Change in alcohol consumption (TLFB)	Recruiting 2025–2028
A Study of brenipatide in participants with alcohol use disorder	Lilly pharmaceuticals	Mild AUD *N* = 1100	NCT07219953 Worldwide (115 sites) Eli lilly and company	Brenipatide sc vs. placebo 56 weeks	Change in alcohol consumption (TLFB)	Recruiting 2025–2028
GLP‐1 Receptor agonists to decrease ethanol and CVD risk in HIV	Tindle et al.	AUD and HIV‐1 BMI: ≥ 23 kg/m^2^ *N* = 200	NCT07221214 USA (Nashville, TN) Vanderbilt university medical center	Oral semaglutide (Rybelsus) vs. Placebo 3 months	Average drinks/week past 30 days at 3 months (TLFB)	Not yet recruiting 2026–2029
Semaglutide (SEMA) for alcohol use disorder (AUD) after metabolic and bariatric surgery (MBS)	Ivezaj et al.	AUD and laparoscopic roux‐en‐Y gastric bypass or sleeve gastrectomy BMI: ≥ 30 and < 50 kg/m^2^ *N* = 10	NCT07223983 USA (New Haven, CT) Yale university	Semaglutide sc 0.25–1 mg weekly vs. wait‐list control 3 months	Percent weight change; drinks per drinking day; drinks per calendar day; heavy drinking days; drinking vs. abstinent days	Not yet recruiting 2026
Combination therapy for alcohol use disorder	Huhn et al.	AUD BMI: ≥ 18.5 kg/m^2^ *N* = 45	NCT07249554 USA (Baltimore, MD) Johns hopkins university	Placebo + placebo vs. Placebo + GLP‐1 vs. GLP‐1 + Naltrexone 14 days	Participant‐reported adverse events	Not yet recruiting 2026–2028
Tirzepatide combined with cognitive‐behavioral therapy (CBT) for adults with alcohol use disorder (AUD) and overweight/obesity (OOB)	Morley et al.	AUD BMI: ≥ 27 kg/m^2^ *N* = 46	NCT07292519 New south wales, australia South western sydney local health district	Tirzepatide sc vs. placebo 12 weeks	Change in heavy drinking days (TLFB)	Not yet recruiting 2026–2028
The Effects of exenatide, a GLP‐1 agonist, on alcohol self‐administration in heavy drinkers	Devine et al.	AUD BMI: ≥ 18 and < 30 kg/m^2^ *N* = 8	NCT03645408 USA (Boston, MA) Boston medical center	Exenatide vs. placebo (within‐subjects) 2 days	Alcohol consumption (2 h)	Terminated 2019–2021

Abbreviations: AUD, alcohol use disorder; BMI, body mass index; DPP‐4I, dipeptidyl peptidase‐4 inhibitor; GLP‐1RA, glucagon‐like peptide‐1 receptor agonists; sc, subcutaneous; T2D, type‐2 diabetes; TLFB, Timeline Followback.

^a^ Data from completed studies here have not yet been published or posted on clinicaltrials.gov.

A 16‐week study that has not yet begun recruiting (NCT06546384) will examine the effects of semaglutide among 64 patients with obesity and hepatic steatosis who have an AUDIT‐C score reflecting harmful drinking. Participants will be randomly assigned to receive semaglutide at a maximum dosage of 2.4 mg/week or counseling only. The study's primary outcome will be the proportion of patients who achieve total alcohol abstinence confirmed by a negative phosphatidylethanol test. A planned human laboratory study (NCT07249554) will combine semaglutide with naltrexone in 45 patients with AUD to assess the safety and efficacy of the combination. Patients will be recruited from an inpatient AUD treatment program and randomly assigned to receive double placebo, oral semaglutide + placebo, or oral semaglutide + naltrexone for 2 weeks. The dosage of semaglutide will be 3 mg daily for the first week and up to 7 mg daily during week 2. During study visits, patients will complete assessments of alcohol craving, alcohol demand, anhedonia, eating behaviors, and subjective effects. A 3‐month trial that has not yet begun to recruit participants (NCT07221214) will investigate the efficacy of oral semaglutide (dosage unspecified) in reducing weekly drinks/week in 200 individuals living with HIV who currently consume alcohol at a non‐harmful level. Patients will be randomly assigned to receive oral semaglutide or placebo with the primary outcome being a reduction in mean drinks/week. Another study will investigate the impact of semaglutide 1 mg/week on an unspecified number of individuals with AUD who have undergone laparoscopic Roux‐en‐Y gastric bypass or sleeve gastrectomy (NCT07223983), but who have obesity or are overweight and have a medical comorbidity. Patients will be randomly assigned to semaglutide or a wait‐list control for 3 months with a subsequent 6‐month follow‐up. The primary outcomes of this study are weight loss and decreases in four measures of drinking. Finally, a multicenter study in 622 US veterans with AUD has not yet begun recruitment. It will be a double‐blind, placebo‐controlled, 28‐week RCT examining the safety and efficacy of semaglutide, up to a maximum of 2.4 mg/week (NCT07218354). The primary outcome will be a two‐level reduction in the World Health Organization risk drinking level from baseline.

Numerous studies of multiple‐action incretin‐based drugs' effects on alcohol consumption are in progress. A proof‐of‐concept 24‐week clinical trial (NCT06987513) for which recruitment was completed, randomly assigned 100 patients with AUD to receive a maximum weekly dose of 2.4 mg of the GLP‐1/glucagon dual receptor agonist pemvidutide or placebo. That study is expected to conclude in September 2026. A 28‐week placebo‐controlled trial of mazdutide (NCT06817356), a dual GLP‐1/GIP receptor agonist (at an unspecified dosage) completed enrollment of 300 patients with AUD and is expected to conclude in August 2028. An 8‐week placebo‐controlled trial of tirzepatide (NCT06994338), a dual GLP‐1/GIP receptor agonist, at a maximum weekly dosage of 5 mg is currently recruiting 42 participants with moderate or severe AUD, with a primary outcome being the number of HDDs during the last 4 weeks of treatment. The study is expected to conclude in August 2026. A 4‐week study, the primary aim of which is to determine the effects of tirzepatide on cue‐reactivity among 20 individuals with AUD (NCT06727331) is anticipated for completion in July 2026. A planned 8‐week, placebo‐controlled study of tirzepatide, up to 5 mg/week, will recruit 46 participants with AUD and overweight/obesity (NCT07292519). Finally, two phase 3 studies of brenipatide, a dual GLP‐1RA/GIP agonist, are enrolling participants with AUD. Each 56‐week study will enroll 1100 participants recruited at > 100 sites internationally. The first study (NCT07219953) is recruiting individuals with mild AUD, while the second (NCT07219966) is recruiting individuals with moderate‐to‐severe AUD. The primary outcome measure in both studies is “a change in drinking patterns.”

## 4 Future Directions and Barriers for GLP‐1RAs in Treating SUDs

Although most of the substance‐related research with GLP‐1RAs has focused on their alcohol‐related effects, their impact on the use of other substances—including opioids, nicotine, and psychostimulants—has also been evaluated. Several animal studies have shown that exendin‐4 and liraglutide treatment attenuate heroin, fentanyl, and morphine self‐administration (Klausen, Thomsen, et al. 2022). Similarly, preclinical studies demonstrate that GLP‐1RAs reduce nicotine self‐administration and dampen the rewarding and locomotor‐stimulating effects of psychostimulants like cocaine and amphetamine (Hernandez and Schmidt 2019; Egecioglu et al. 2013b, 2013a). A protective effect of GLP‐1RAs was also seen in overdose risk among individuals with opioid use disorder (OUD) (aIRR = 0.60 [0.43–0.83]) (Qeadan et al. 2025). Another EHR‐based study showed that the use of GLP‐1/GIP dual‐action agonists is associated with reduced risk for OUD (HR = 0.87), cannabis use disorder (HR = 0.88), and stimulant use disorder (HR = 0.84), suggesting a potential protective effect of GLP‐1RAs across substances (Xie et al. 2025). Given these promising findings, which support the potential for targeting shared neurobiological and reward pathways across SUDs, more RCTs are required to evaluate the use of GLP‐1RAs for treating AUD and other SUDs.

Despite their promise, several factors could limit the broader clinical application of GLP‐1RAs. These include adverse GI side effects such as nausea and vomiting (Gorgojo‐Martinez et al. 2022), especially with dose escalation, and the inconvenience or aversion to subcutaneous administration despite a growing number of oral formulations (Duncanson et al. 2021), both of which appear to reduce treatment adherence. The high cost of GLP‐1RAs, given the absence of generic versions, also limits their availability (Gleason et al. 2024).

An oral formulation of semaglutide, approved for treating T2D, was recently FDA approved for treating overweight/obesity (Novo Nordisk 2025) and could improve adherence (Lutkemeyer et al. 2024). Clinical trials of oral semaglutide for treating T2D show that it is also efficacious in patients with comorbid conditions, including moderate renal impairment (Mosenzon et al. 2019). Orforglipron, a once‐daily oral non‐peptide, small‐molecule GLP‐1RA was recently FDA approved for weight loss (U.S. Food and Drug Administration 2026a). The medication can be taken without food or water restrictions. There are both advantages and disadvantages to both the oral and subcutaneous routes of administration (Meier 2021; Formichi et al. 2024). Recent approval of a 7.2 mg dose of semaglutide may also have implications for treating AUD (U.S. Food and Drug Administration 2026b). As more formulations of GLP‐1RAs become available, clinicians will have more options to select the formulation that best matches their patients' needs and preferences.

In addition to the standard approach of gradually increasing the dosage of GLP‐1RAs to minimize adverse effects, the opposite approach—microdosing with a very gradual escalation in dosage—may be useful in managing GI adverse effects. These advances in GLP‐1RA treatment strategies may increase their use and effectiveness, features that may be of particular importance in treating patients with AUD, whose treatment adherence is often lower than that in other patient populations (Grodensky et al. 2012). Other key considerations are whether, among people with AUD, oral GLP‐1RAs are more acceptable and promote greater adherence than subcutaneous injections, and how to exploit the growth in available GLP‐1RAs to select the optimal candidate drugs for specific individuals. An important question relevant to adherence and overall safety is the extent to which the commonly observed GI adverse events associated with GLP‐1RAs (Jalleh et al. 2026) are additive or multiplicative with those commonly produced by chronic heavy alcohol consumption (Bode and Bode 1997).

Although precision medicine approaches to stratifying patients, including pharmacogenetic studies of both single nucleotide variants and polygenic risk scores, have been explored as predictors of treatment response for T2D and obesity, they have not yielded robust findings (Klen and Dolzan 2022; German et al. 2025). Whereas the effects of GLP‐1RAs in reducing alcohol consumption may be mediated by the same pharmacological effects as in T2D and obesity, other approaches (e.g., proteomics) may be necessary for the biological stratification of patients with AUD prior to starting treatment with a GLP‐1RA.

## Disclosure

Dr. Kranzler is a member of advisory boards for Altimmune and Clearmind Medicine; a consultant to Sobrera Pharmaceuticals, Altimmune, Lilly, Ribocure, and Boehringer‐Ingelheim; and the recipient of research funding and medication supplies for an investigator‐initiated study from Alkermes and company‐initiated studies by Altimmune and Lilly.

## Conflicts of Interest

The authors declare no conflicts of interest.

## Data Availability

Data sharing not applicable to this article as no datasets were generated or analysed during the current study.
